# Stewart Treves syndrome as a catastrophic sequela of chronic lymphedema: Clinical spectrum, management challenges, and outcomes

**DOI:** 10.1016/j.jvscit.2026.102191

**Published:** 2026-02-26

**Authors:** Yasser M. Elkiran, Nshaat A. Elsaadany, Yasmin Y. Elkiran, Ahmad Elghamrey, Mohamed E. Eldin, Ahmed M. Morshed, Ahmed A. Elshabrawy

**Affiliations:** Cardiothoracic and Vascular Surgery Center, Mansoura University, Mansoura, Egypt

**Keywords:** Stewart Treves syndrome, Angiosarcoma, Chronic lymphedema, Postmastectomy, Limb disarticulation

## Abstract

**Objective:**

Stewart Treves syndrome (STS) is a rare, aggressive lymphangiosarcoma arising in chronically lymphedematous tissue, most often after mastectomy with axillary lymph node dissection, and occasionally in lower limb lymphedema. We aimed to describe the clinical spectrum, diagnostic challenges, management, and outcomes of upper and lower limb STS, highlighting the importance of early recognition in improving survival.

**Methods:**

We conducted a retrospective review of three patients diagnosed with STS between 2020 and 2025 at our tertiary vascular surgery center specializing in lymphedema. Clinical presentation, imaging, histopathology, management strategies, and postoperative outcomes were analyzed.

**Results:**

Two female patients developed upper limb STS after breast cancer surgery and radiotherapy, and one male patient developed lower limb STS postcystectomy and pelvic radiotherapy. All presented with violaceous or nodular lesions on chronically lymphedematous limbs, initially misdiagnosed as cellulitis or ecchymosis. Biopsy confirmed angiosarcoma (CD31/CD34 positive). Two patients underwent radical limb disarticulation; one achieved 2-year recurrence-free survival with adjuvant therapy, and the other remained disease-free at 6 months. The third patient, who declined early biopsy, developed rapid pulmonary metastases and died within months.

**Conclusions:**

STS should be suspected in any patient with chronic lymphedema who develops new violaceous, nodular, or ulcerative lesions, particularly in the proximal segments. Prompt biopsy and radical surgical excision remain essential, although the overall prognosis is poor. Prevention through effective lymphedema management, clinician vigilance, and long-term follow-up is critical to decrease this devastating complication.

**Article Highlights**

**Type of Research:** Single-center retrospective case series
**Key Findings:** Three cases of Stewart Treves Syndrome were identified, two in postmastectomy upper limb lymphedema and one in lower limb lymphedema following pelvic surgery and radiotherapy. Radical disarticulation achieved local control in two patients, while one patient who deferred early biopsy developed metastatic disease and died within months.
**Take Home Message:** Stewart Treves Syndrome is a rare but lethal vascular malignancy arising in long-standing lymphedema. Early biopsy of any new violaceous or nodular lesion, chiefly in the proximal portions with subsequent radical excision, is critical to improve outcomes. Proactive lymphedema management and long-term surveillance are essential preventive measures.

Stewart Treves syndrome (STS) is a rare, highly aggressive vascular malignancy with approximately 400 cases reported worldwide in the literature.^1^ Specifically, it is a lymphangiosarcoma that arises within chronically lymphedematous tissue. Initially described by Stewart and Treves in 1948 in women who developed upper limb lymphedema after radical mastectomy and axillary lymph node dissection, since then, STS has been reported in a wider spectrum of primary and secondary lymphedema, including lower limb involvement. Although uncommon, its recognition is critical owing to its aggressive nature and poor prognosis.^2^

Clinically, STS presents with a range of cutaneous manifestations, including violaceous plaques, nodules, erythematous patches, papillomatous lesions, and ulcerations. These features often mimic benign conditions such as cellulitis, ecchymosis, or other vascular lesions, frequently resulting in delayed diagnosis. Definitive diagnosis requires histopathological confirmation, supported by immunohistochemical staining for endothelial markers such as CD31, CD34, and factor VIII-related antigen. Surgical excision, often in the form of limb amputation or disarticulation, remains the mainstay of treatment, with chemotherapy and radiotherapy serving primarily as adjunctive modalities.^3^

This report describes three cases of STS involving both upper and lower limbs, emphasizing the clinical spectrum, diagnostic pitfalls, and treatment outcomes, with a focus on early recognition and multidisciplinary management.

## Methods

### Study design and setting

A retrospective case series was conducted at a tertiary referral vascular surgery center specializing in lymphedema management between 2020 and 2025. Institutional review board approval was waived owing to the retrospective nature of the study and the absence of identifiable patient information. Written informed consent for publication was obtained from all patients (or their legal representatives).

### Inclusion criteria and data collection

Three patients (2 females and 1 male) with mean age of 57.7 years, presented with angiosarcoma (lymphangiosarcoma) as a complication of chronic lower and upper limb lymphedema were included in the study (Table). Diagnosis was confirmed using histopathological assessment of angiosarcoma. Lymphedema history, demographic details, treatment modalities, and survival outcomes were analyzed for the three patients. Patients were followed after surgery to assess wound healing and survival.

**Table:** Summary of clinical characteristics and outcomes

Characteristics	Case 1	Case 2	Case 3
Age and sex	49 female	54 female	70 male
Affected body part	Right upper limb	Left upper limb	Left lower limb
Cause of lymphedema	Right mastectomy and radiotherapy 15 years ago.	Left breast conservative surgery accompanied by radiotherapy 10 years ago.	A radical cystectomy and urinary diversion and postoperative radiotherapy 16 years ago.
Interval between surgery and lymphedema	5	2	1
Duration of lymphedema	10	8	15
Adjuvant radiotherapy	+	+	+
Presentation	Scattered spontaneous violaceous patches.	Erythematous patches.	skin color changes, skin papillomatosis and bleeding ulcers.
Management	Left upper limb disarticulation	Palliative chemotherapy Pain control therapy	Left lower limb disarticulation
Outcome	Recurrence-free survival at 2 years	Died within a few months.	Cancer-free wound edges with no metastases on positron emission tomography with computed tomography after 6 months follow up.

## Results

### Case 1

**Fig 1:**
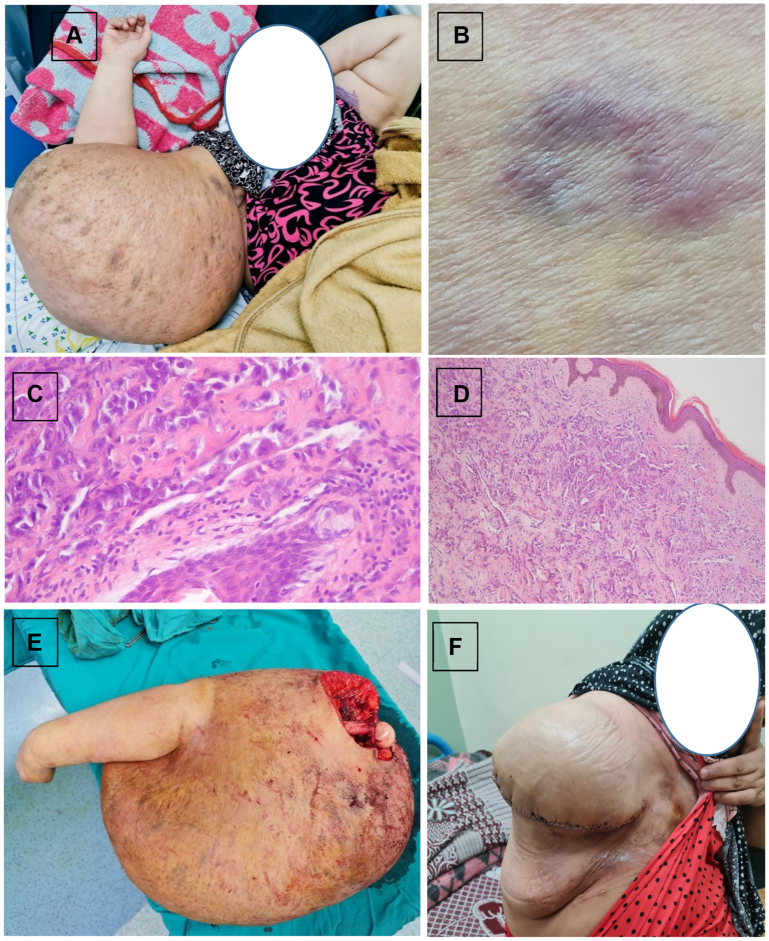
Case 1. Huge lymphedema (localized to the right upper limb) post mastectomy **(A)** with violaceous plaques **(B)**. Histopathology showing anastomosing vascular channels lined by atypical endothelial cells with multilayered proliferation, nuclear pleomorphic, and moderate mitotic activity **(C and D)**. Disarticulation of right upper arm with 33 kg lymphedematous tissue **(E and F)**.

A 49-year-old woman who had undergone right mastectomy and adjuvant radiotherapy 15 years earlier subsequently developed chronic right upper-limb lymphedema 5 years later. After a decade of persistent lymphedema, she presented with localized swelling of the right upper limb (Fig 1, *A*) and violaceous plaques (Fig 1, *B*), initially misinterpreted clinically as ecchymotic patches. A biopsy was obtained immediately upon the patient's initial presentation to our center, approximately 3 weeks after the initial appearance of the cutaneous lesion and revealed low-grade angiosarcoma (lymphangiosarcoma) with immunohistochemistry positive for CD31 and CD34, anastomosing vascular channels lined by atypical endothelial cells with multilayered proliferation, marked nuclear pleomorphism, and moderate mitotic activity (Fig 1, *C* and *D*). Multidisciplinary evaluation recommended definitive surgical management, and the patient underwent right upper-limb disarticulation with removal of approximately 33 kg of lymphedematous tissue (Fig 1, *E* and *F*). Preoperatively, the massive limb burden caused significant difficulty in walking, impaired coordination, and severe functional limitations. Postoperatively, the patient reported substantial improvement in mobility and balance, expressing satisfaction with her restored ability to walk comfortably and perform daily activities. She remains recurrence free at 2 years of follow-up, highlighting the effectiveness of timely recognition and aggressive management of STS.

### Case 2

**Fig 2:**
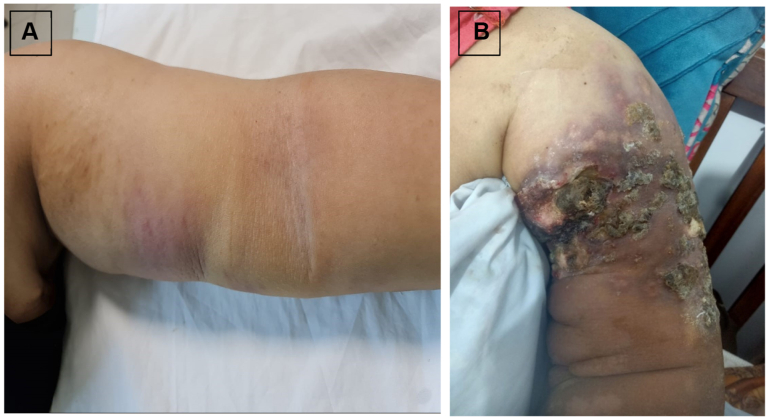
Case 2. Erythematous skin patches over the volar aspect of the chronically lymphedematous left upper limb **(A)**, initially misdiagnosed and treated as cellulitis without improvement. Rapidly progressive ulcerative lesions **(B)** that developed three months later after the patient declined initial biopsy, ultimately diagnosed as high-grade angiosarcoma.

A 54-year-old woman underwent left breast-conserving surgery followed by adjuvant radiotherapy 10 years earlier. She developed chronic left upper limb lymphedema 2 years postoperatively and later presented with erythematous skin patches over the volar aspect of the chronically lymphedematous left upper limb (Fig 2, *A*), which were initially treated as cellulitis without clinical improvement. She declined biopsy at that time despite being informed of possible complications and re-presented 3 months later with rapidly progressive ulcerative lesions (Fig 2, *B*), at which time histopathological evaluation confirmed high-grade angiosarcoma (lymphangiosarcoma). Subsequent positron emission tomography with computed tomography demonstrated pulmonary metastases. Despite supportive management, the patient deteriorated quickly and after multidisciplinary evaluation, the patient was declared unfit for surgical intervention. The patient was headed toward palliative therapy; she developed respiratory failure, and died shortly thereafter, underscoring the aggressive nature of radiation-associated angiosarcoma and the critical importance of early biopsy in nonresolving cutaneous changes in lymphedematous limbs.

### Case 3

**Fig 3:**
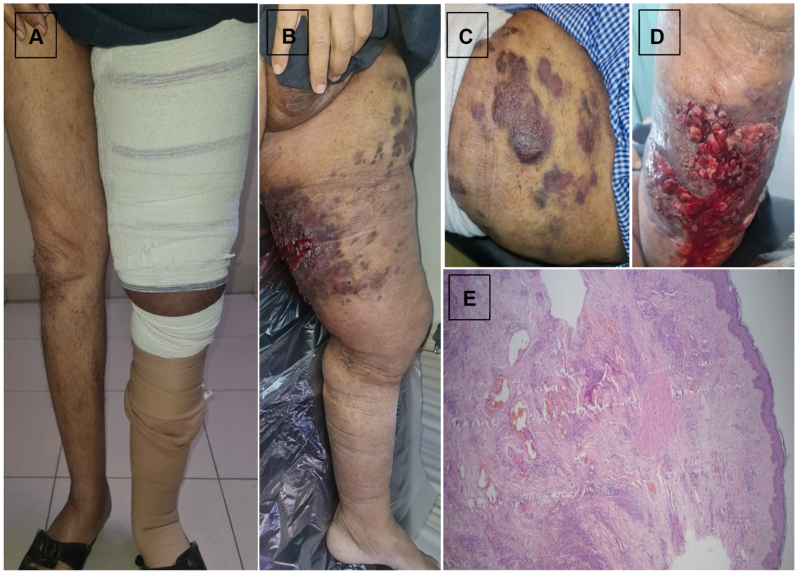
Case 3. Chronic left lower limb lymphedema with progressive swelling **(A)**. Violaceous plaques anterior upper part of the left thigh **(B and C)**. Ulcerated bleeding papillomatous lesion at back of the midthigh **(D)**. Histopathology showing features consistent with angiosarcoma (Stewart-Treves syndrome [STS]), including anastomosing vascular channels lined by atypical endothelial cells **(E)**.

A 70-year-old man underwent radical cystectomy and postoperative radiotherapy 16 years earlier, followed by chronic left lower limb lymphedema for 15 years. He presented with progressive swelling (Fig 3, *A*) violaceous plaques anterior upper part of the left thigh (Fig 3, *B* and *C*), and ulcerated bleeding papillomatous lesions at the back of the midthigh (Fig 3, *D*). A biopsy was performed promptly upon presentation, approximately 6 weeks after the initial appearance of the cutaneous lesion. Histopathology showed features consistent with angiosarcoma (STS), including anastomosing vascular channels lined by atypical endothelial cells (Fig 3, *E*). After a multidisciplinary evaluation, left lower limb disarticulation was performed. He remained recurrence free with cancer-free margins at 6 months.

## Discussion

STS remains a rare but formidable malignancy arising in the context of chronic lymphedema. Although historically associated with postmastectomy upper limb lymphedema, it has now been observed after a variety of insults, including pelvic surgery, radiation therapy, trauma, chronic venous insufficiency, and infectious etiologies. The pathogenesis of STS is closely linked to chronic lymph stasis, tissue hypoxia, persistent inflammation, and impaired immune surveillance. These conditions create a proangiogenic microenvironment that facilitates malignant transformation of endothelial cells. The latency period between lymphedema onset and tumor development is variable, but typically spans 5 to 20 years, reflecting the slow but insidious process of malignant transformation in chronically compromised lymphatic tissue.^3^^,^^4^

The differential diagnosis for STS includes both benign and malignant vascular or skin lesions in lymphedematous limbs. Kaposi sarcoma can mimic STS, but is positive for human herpesvirus 8, unlike STS. Metastatic breast carcinoma and infectious lesions such as cellulitis or erysipelas must also be excluded. Other considerations include lymphangioendothelioma, hemangioma, melanoma, pyogenic granuloma, and squamous cell carcinoma. The diagnosis is confirmed via biopsy with immunohistochemistry, while imaging helps to assess local extent and staging.^5^^,^^6^ The pathophysiological hallmark of STS is the chronic lymphostatic environment, which impairs local immune surveillance, promotes persistent inflammation, and drives proangiogenic signaling, ultimately predisposing to endothelial malignancy. The latency period observed in our cases, ranging from 10 to 15 years, aligns with published data reporting a 5- to 20-year interval between lymphedema onset and sarcoma development, underscoring the need for lifelong surveillance in affected individuals.^7^

Phenotypically, STS presents with variable cutaneous lesions that often mimic benign inflammatory or vascular conditions. In our series, initial misdiagnosis occurred in two patients, whose early lesions were interpreted as cellulitis or ecchymosis, a scenario widely adopted in dermatologic practice, where none of the patients had received structured or effective long-term lymphedema management before referral, and limited adherence or delayed access to specialized care contributed to advanced disease at presentation. All three patients were evaluated at our center for the first time with established clinical features of STS, highlighting the critical role of early referral and comprehensive lymphedema therapy in preventing malignant transformation. Imaging modalities such as computed tomography, magnetic resonance imaging, and positron emission tomography with computed tomography can aid in evaluating the extent of local disease and detecting metastasis, but they lack specificity to reliably differentiate STS from lymphedema-related tissue changes. Consequently, tissue biopsy remains essential for definitive diagnosis, with histopathology and immunohistochemistry providing the critical confirmation of malignant endothelial proliferation.^8^^,^^9^

In terms of management, radical surgical excision with wide margins frequently necessitating limb amputation or disarticulation remains the only intervention with a meaningful impact on local disease control, while hyperthermic isolated limb perfusion has been explored in selected cases of extremity angiosarcoma; however, its role in STS remains undefined owing to the paucity of evidence and the aggressive biological behavior of this malignancy.^9^ In our series, two patients underwent major limb disarticulation and achieved temporary remission, reporting improved mobility and functional outcomes after removal of the bulky, edematous limb. Adjunctive chemotherapy and radiotherapy may provide symptomatic relief or marginally improve local control, but they do not substantially alter overall survival, consistent with current evidence positioning these modalities as secondary rather than curative measures.^10^^,^^11^

In all three cases, STS developed in the proximal segments of the affected limbs, specifically the upper arm in postmastectomy lymphedema and the proximal thigh in lower limb involvement. This proximal predilection likely reflects the regions with the most severe, long-standing lymphatic stasis and tissue hypoxia, which create a microenvironment conducive to malignant endothelial transformation. Clinicians should therefore maintain heightened vigilance for new violaceous or nodular lesions in these proximal regions, because early recognition and biopsy are critical for timely diagnosis and improved outcomes. Despite aggressive surgical intervention, the prognosis of STS remains poor, with 5-year survival rates consistently reported to be <20%,^12^^,^^13^ largely owing to high recurrence, rapid systemic dissemination, and diagnostic delays, exemplified in our series by the patient who declined early biopsy and rapidly developed metastatic disease. These findings underscore the importance of prompt, coordinated multidisciplinary management; future therapeutic advancements will likely depend on elucidating the molecular pathways driving malignant transformation in chronically lymphedematous tissue and developing targeted interventions to address this highly aggressive malignancy.

### Limitations

The findings of this study should be interpreted in light of inherent methodological limitations, including the small cohort size dictated by the exceptional rarity of STS, the retrospective nature of data collection, and the relatively short follow-up period in one patient. Nevertheless, the presented cases offer clinically relevant observations regarding disease presentation, diagnostic delay, and therapeutic outcomes, thereby contributing novel and practical insights to the scarce literature on this highly aggressive complication of chronic lymphedema.

## Conclusions

STS should be suspected in any patient with chronic lymphedema who develops new violaceous, nodular, or ulcerative lesions, particularly in the proximal segments of affected limbs. Early recognition, prompt biopsy, and radical surgical excision remain essential, although the overall prognosis is poor. Effective prevention through comprehensive lymphedema management, clinician vigilance, and long-term surveillance is critical to mitigate the risk of this rare but highly lethal complication. Maintaining a high index of suspicion for new vascular or violaceous lesions in lymphedematous limbs is vital, as timely intervention can significantly improve outcomes and survival.

## Author Contributions

Conception and design: NA, YY, AM, ME

Analysis and interpretation: AM

Data collection: YM, AM, AA

Writing the article: YM, NA, YY, AM, AA

Critical revision: YM, NA, AM, ME, AM

Approval of the manuscript: YM, NA, YY, AM, ME, AM, AA

Agreement to be accountable: YM, NA, YY, AM, ME, AM, AA

Statistical analysis: NA

## Funding

None.

## Disclosures

None.
